# Supplementary material for: Analysis of Antibiotic Response in Clinical Wound Pseudomonas aeruginosa Isolates: Unveiling Proteome Dynamics of Tobramycin-Tolerant Phenotype
Source: Mol Cell Proteomics. 2024 Oct 16;23(12):100861. doi: 10.1016/j.mcpro.2024.100861 (PMC11617395; doi:10.1016/j.mcpro.2024.100861)
Supplement: Supplementary materials [file mmc1.docx]

Analysis of antibiotic response in Clinical Wound *Pseudomonas aeruginosa* isolates: Unveiling Proteome Dynamics of tobramycin tolerant phenotype

Kasandra Buchholtz, Rosa Jersie-Christensen, Karen Angeliki Krogfelt, Biljana Mojsoska*

Email: [biljana@ruc.dk](mailto:biljana@ruc.dk)

Department of Science and Environment, Roskilde University, Roskilde, Denmark

**Materials and Methods**

**Minimum inhibition concentration**

The minimum inhibitory concentration (MICs) for the clinical wound isolates and PAO1 towards tobramycin was determined following standard MIC protocols described before (Mojsoska et al., 2015). Briefly, the MIC values were determined by incubating the culture for 24 hours at 37°C, (n=3) with various concentrations (tobramycin 32 - 0.05 µg/ml; the inhibitory effect was observed based on the lack of turbidity, Table S1). Following the MIC, when the wound isolates and PAO1 were treated with tobramycin concentrations at 10 x MIC (killing assay), colony forming units (CFU assay) were counted to estimate bacterial survival (Figure S1). The experiment was performed using cationic adjusted Mueller-Hinton Borth (MHBII) (Becton and Dickinson) was used to test the susceptibility to tobramycin.

Table S1. Minimum inhibitory concentration of 3 *Pseudomonas aeruginosa* isolates.

| Isolate | PAO1 | 9000a (patient1) | 989a (patient2) |
| --- | --- | --- | --- |
| MIC (Tobramycin) | 0.4 µg/ml | 0.4 µg/ml | 0.4 µg/ml |


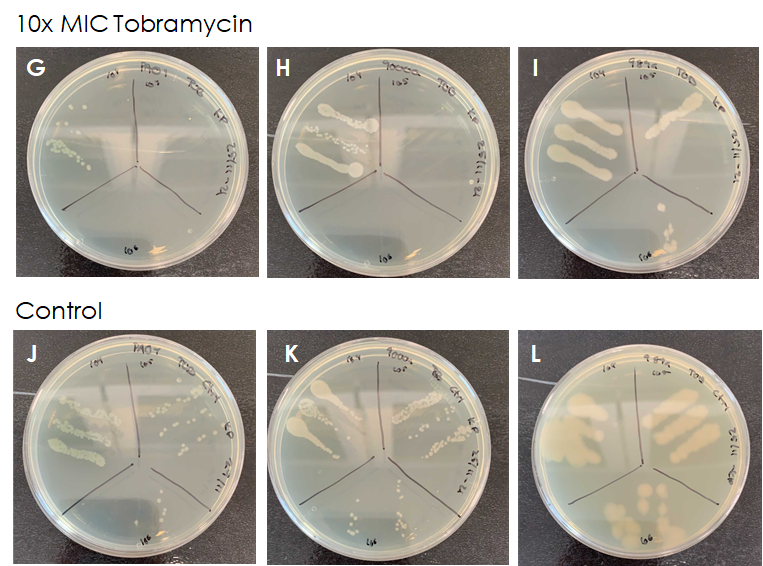


Figure S1. **Spot test analysis**. Colony forming units of tobramycin tolerant test. Two *P. aeruginosa* wound isolates 9000a, and 989a and laboratory standard PAO1 (n = 3), were used for the experiment. The overnight culture was diluted 1:100 in 20 ml cationic adjusted Mueller-Hinton Broth (MHBII) and incubated for 24 hours at 37C°. At the stationary phase, the cultures were split into a control group and a treatment group. The culture was treated with 10xMIC tobramycin. After 24 hours of exposure, the culture was plated on LB agar plate for growth assessment. G) PAO1, H) 9000a, I) 989a, J) PAO1 control, K) 9000a, L) 989a, were all cultured in MHBII and treated with tobramycin. All *P. aeruginosa* strains show tobramycin tolerant profile (CFU/ml>10000).


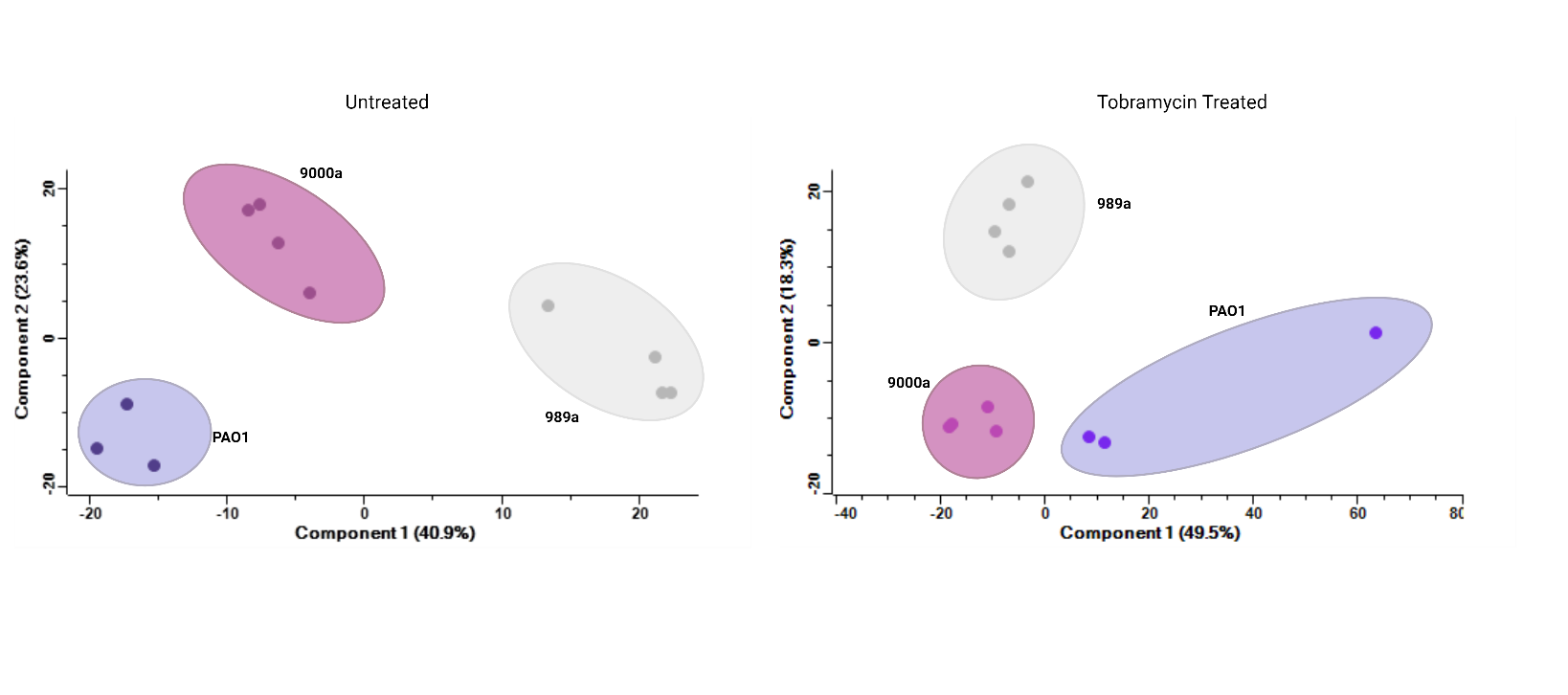


Figure S2. **Principal component analysis (PCA) plots**. ~~A)~~ PCA of untreated (left) and treated (right) samples of the 3 *P. aeruginosa* isolates, PAO1(purple), 989a (grey), and 9000a (pink). Dots represent biological replicates.


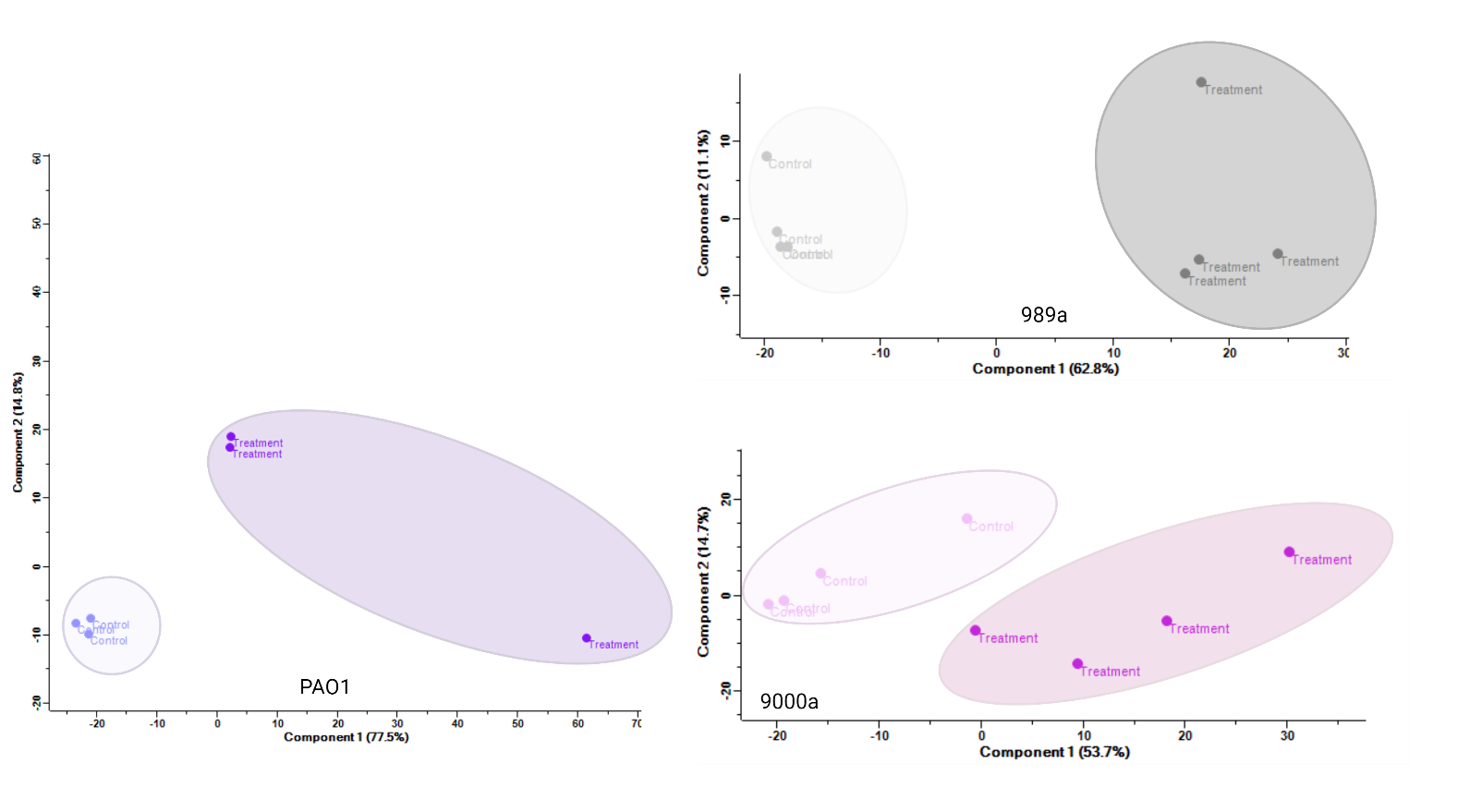


Figure S3 ~~A)~~ **Principal component analysis plots of the individual analysis for *P. aeruginosa* isolates.** PCA for PAO1 (purple), 989a (grey), and 9000a (pink) are shown. The analysis are from tobramycin treatment and the control (no treatment) for each isolate. Dots represent biological replicates.


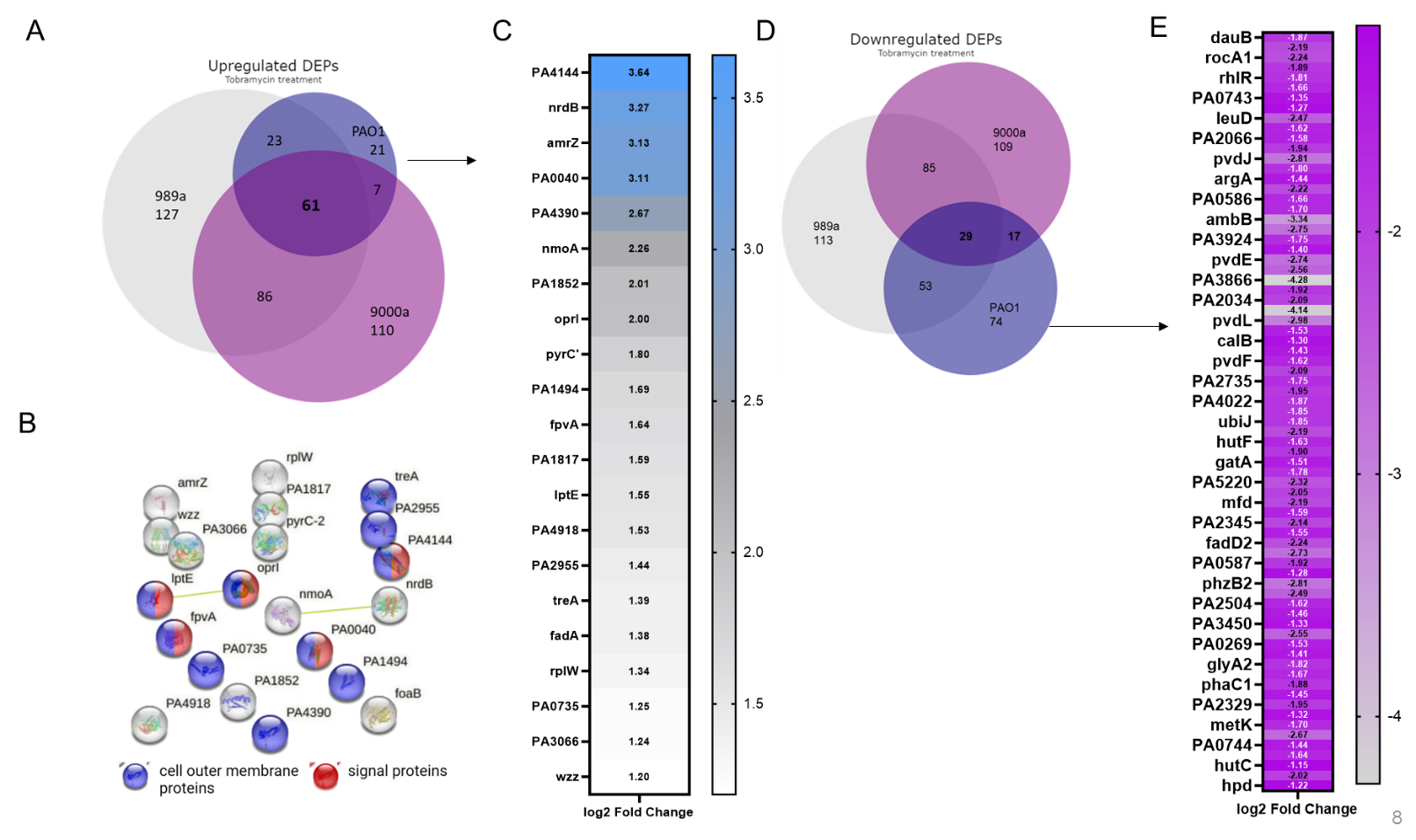


Figure S4. Up- and Down regulated DEPs in PAO1. A) Venn diagram of all differentially expressed proteins (DEPs) that are upregulated in the antibiotic treated *P. aeruginosa* isolates, PAO1, 989a and 9000a. B) Visualization and enrichment of the top proteins was done using STRING (version 12. C) Log2-FC of the 21 upregulated DEPs. D) Venn diagram of all differentially expressed proteins (DEPs) that are downregulated in the antibiotic treated *P. aeruginosa* isolates, PAO1, 989a and 9000a. E) Log2-FC of the 74 downregulated DEPs (log2FC>-1.5).


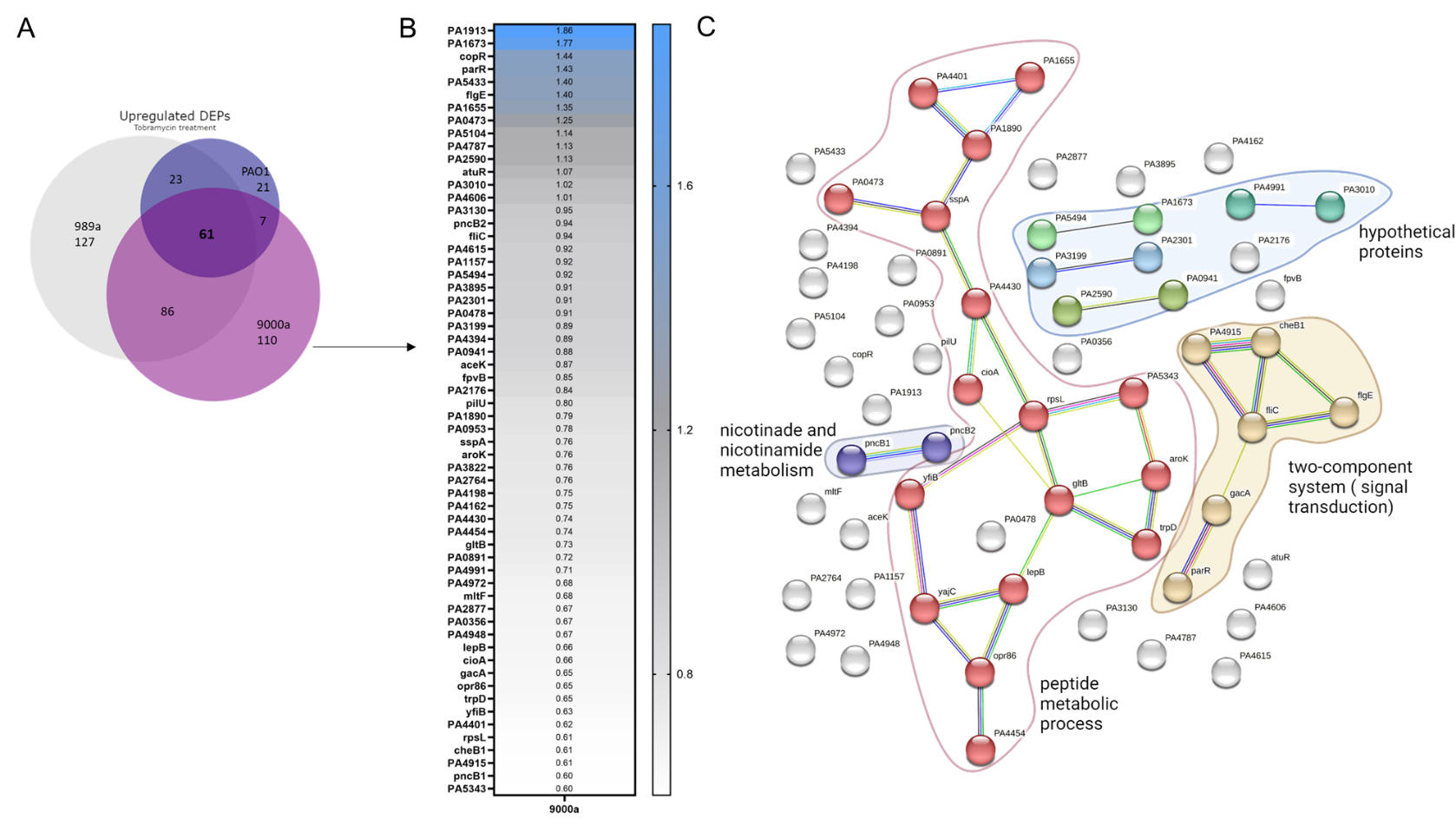


Figure S5. **Upregulated DEPs in isolate 9000a**. A) Venn diagram of all differentially expressed proteins (DEPs) that are upregulated in the antibiotic treated *P. aeruginosa* isolates, PAO1, 989a and 9000a. B) Log2-FC of DEPs found unique for 9000a with log2-FC >0.5. C) Visualization and enrichment of the top proteins was done using STRING (version 12.0).


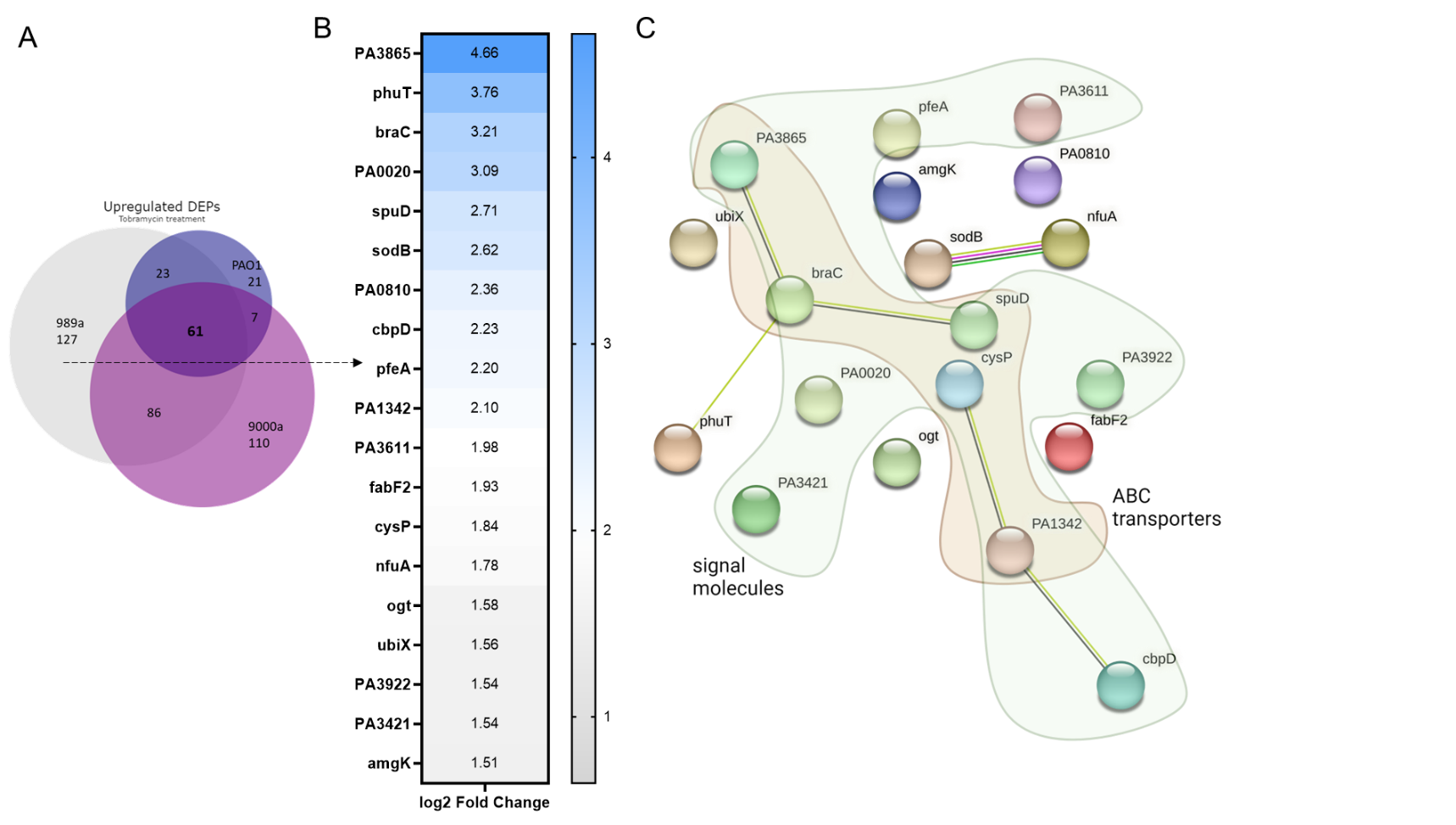


Figure S6. **Upregulated DEPs in isolate 989a** A) Venn diagram of all differentially expressed proteins (DEPs) that are upregulated in the antibiotic-treated *P. aeruginosa* isolates, PAO1, 989a, and 9000a. B) Log2-FC>1.5 of unique DEPs for 989a. C) Visualization and enrichment of the top proteins was done using STRING (version 12.0).


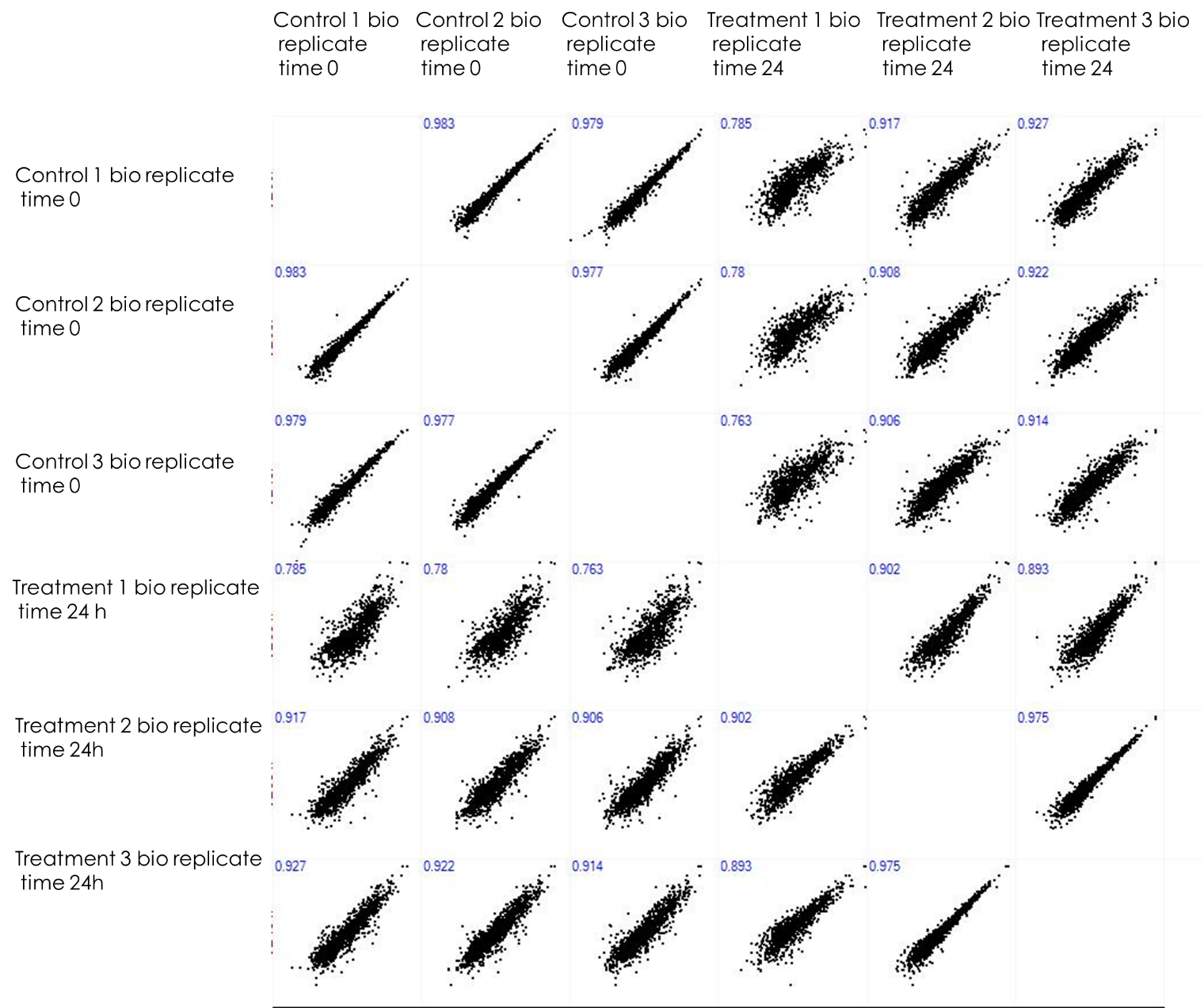


Figure S7. **Multi scatter plots are generated for all biological replicates for the control and treatment samples in isolate PAO1.** The plots generated by Perseus and Pearson correlation values are shown.


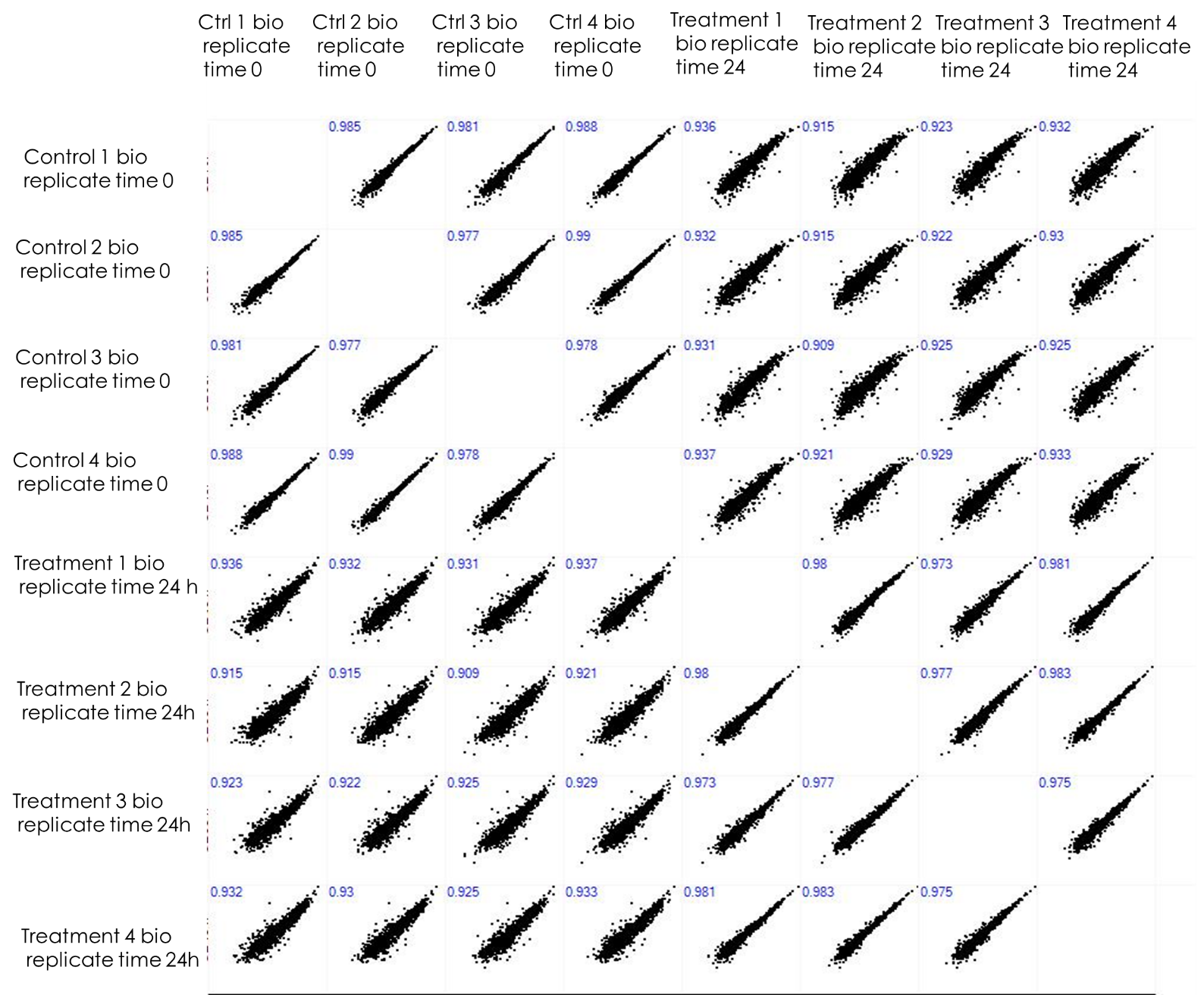


Figure S8. **Multi scatter plots are generated for all biological replicates for the control and treatment samples in isolate 989a.** The plots generated by Perseus and Pearson correlation values are shown.


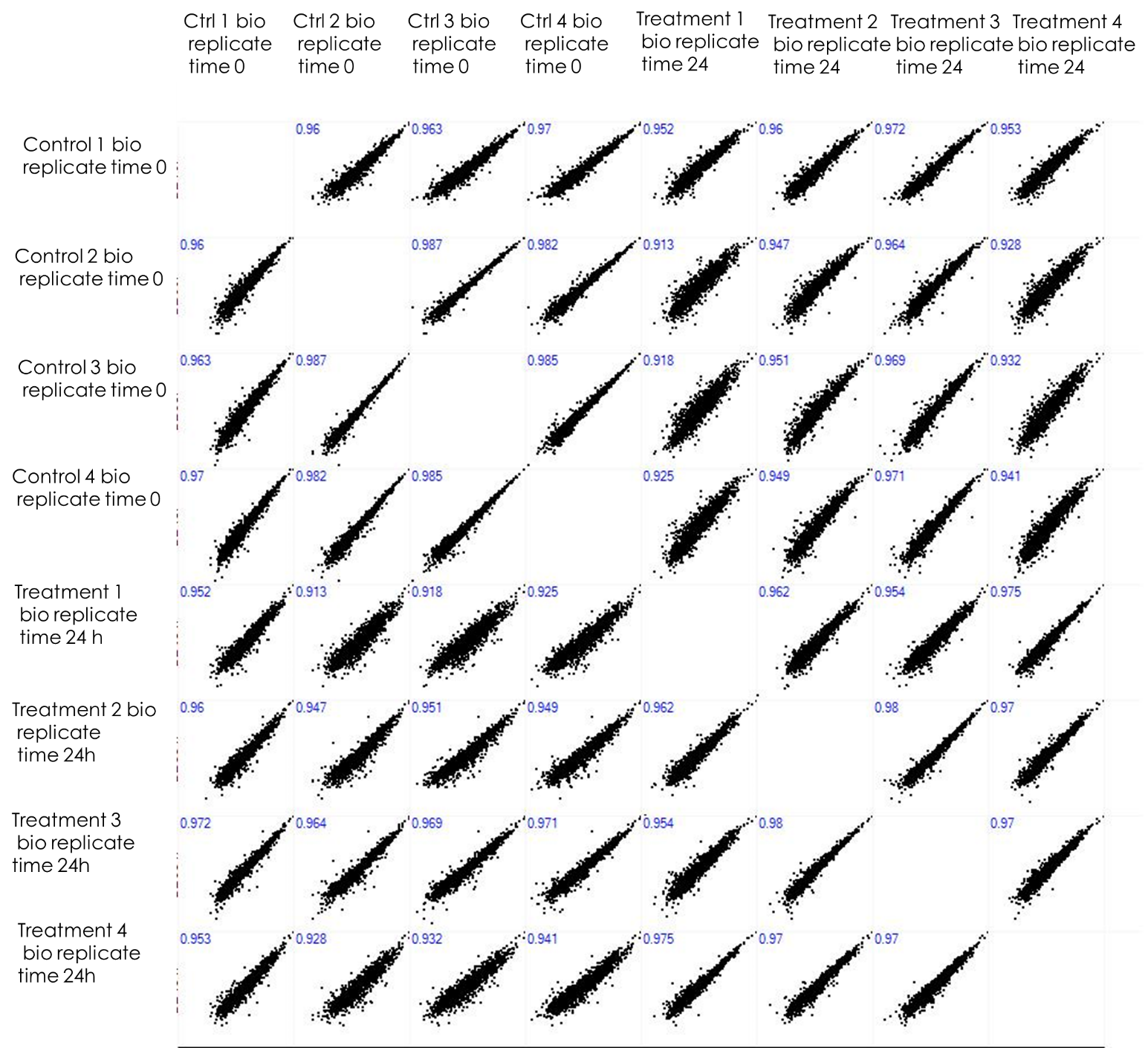


Figure S9. **Multi scatter plots are generated for all biological replicates for the control and treatment samples in isolate 9000a.** The plots generated by Perseus and Pearson correlation values are shown.


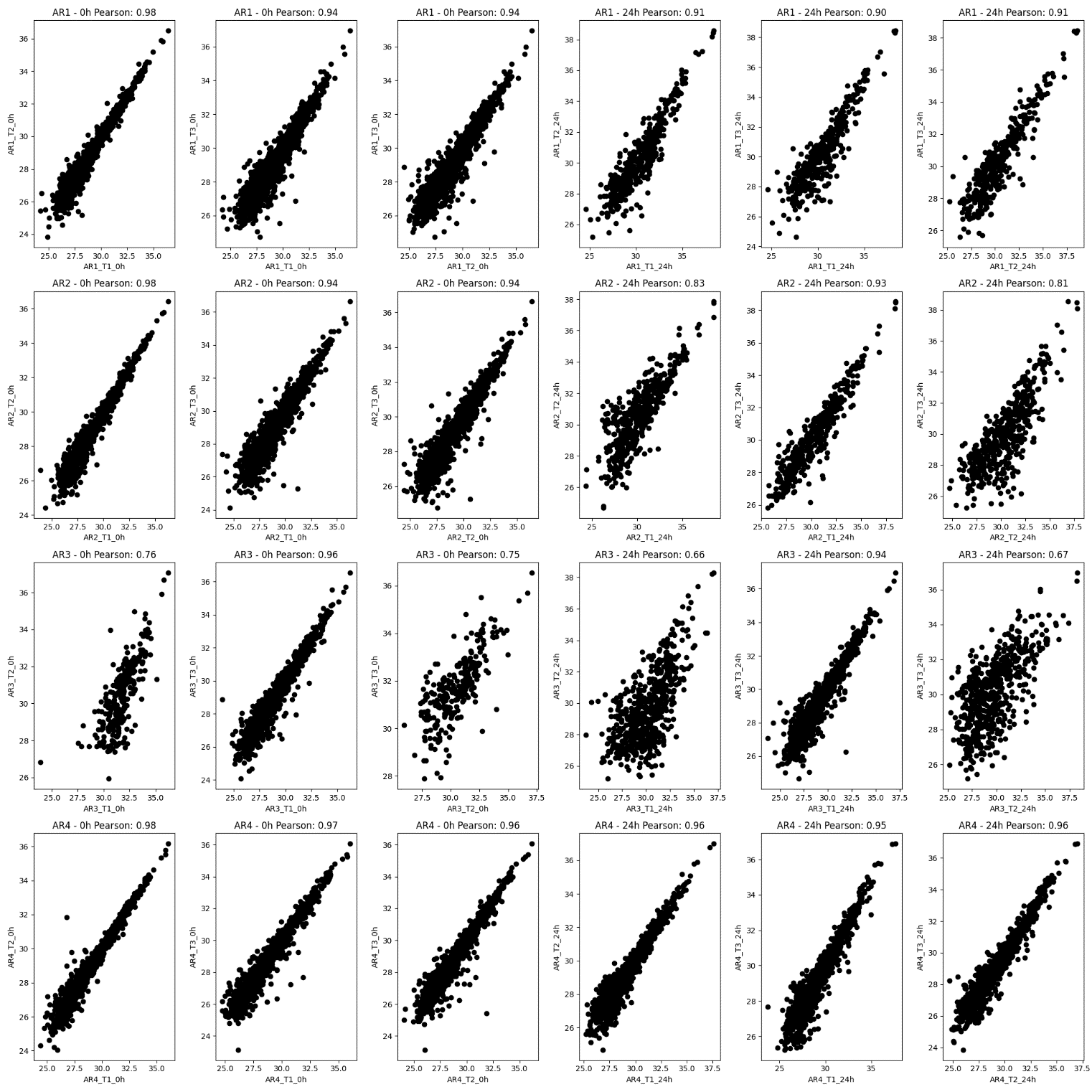
Figure S10. **Multi scatter plots for all technical replicates for the control and treatment samples in isolate PAO1 (AR, T-technical replicate).** The plots are generated by Python and Pearson correlation values are shown.


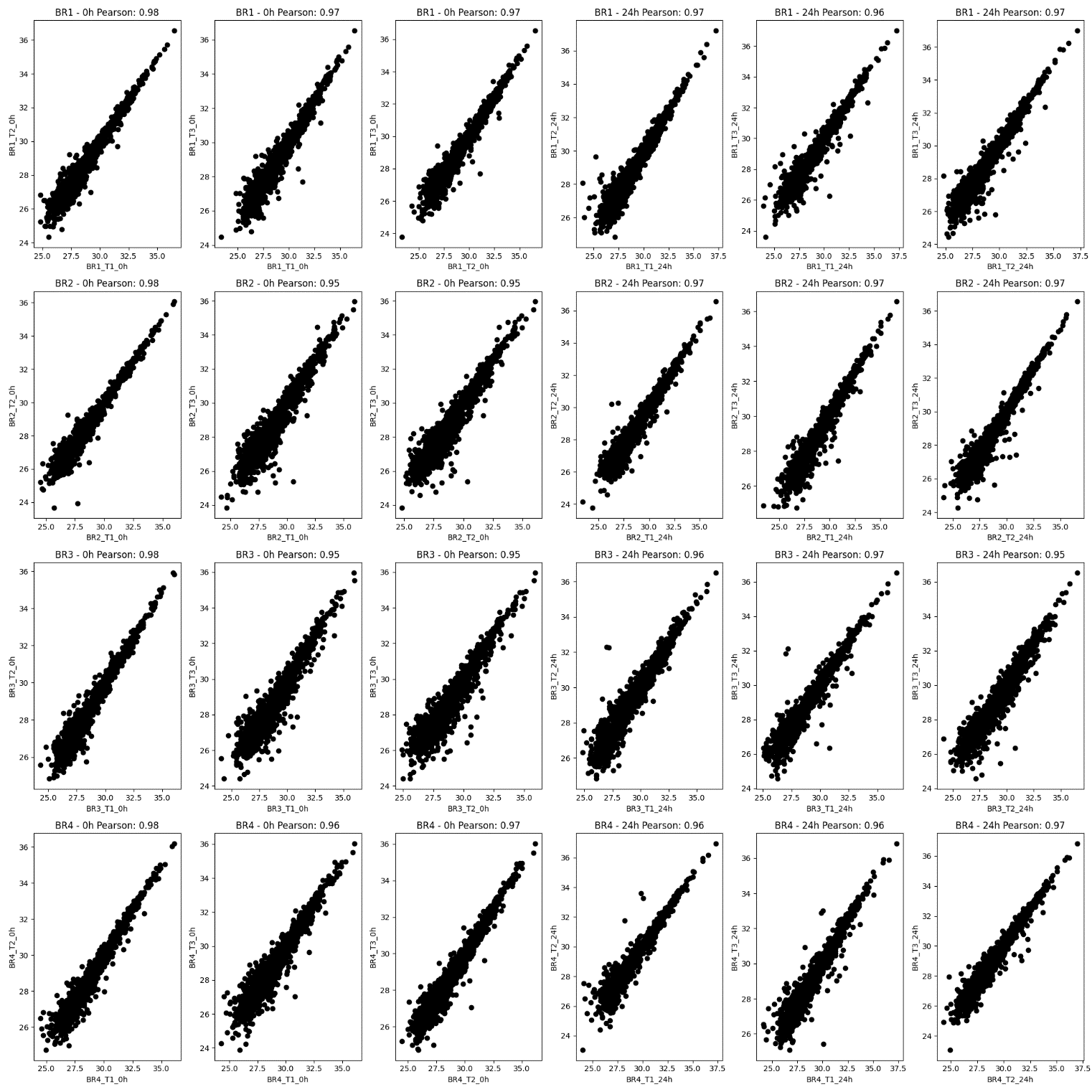


Figure S11. **Multi scatter plots for all technical replicates for the control and treatment samples in isolate 9000a (AR, T-technical replicate).** The plots are generated by Python and Pearson correlation values are shown.


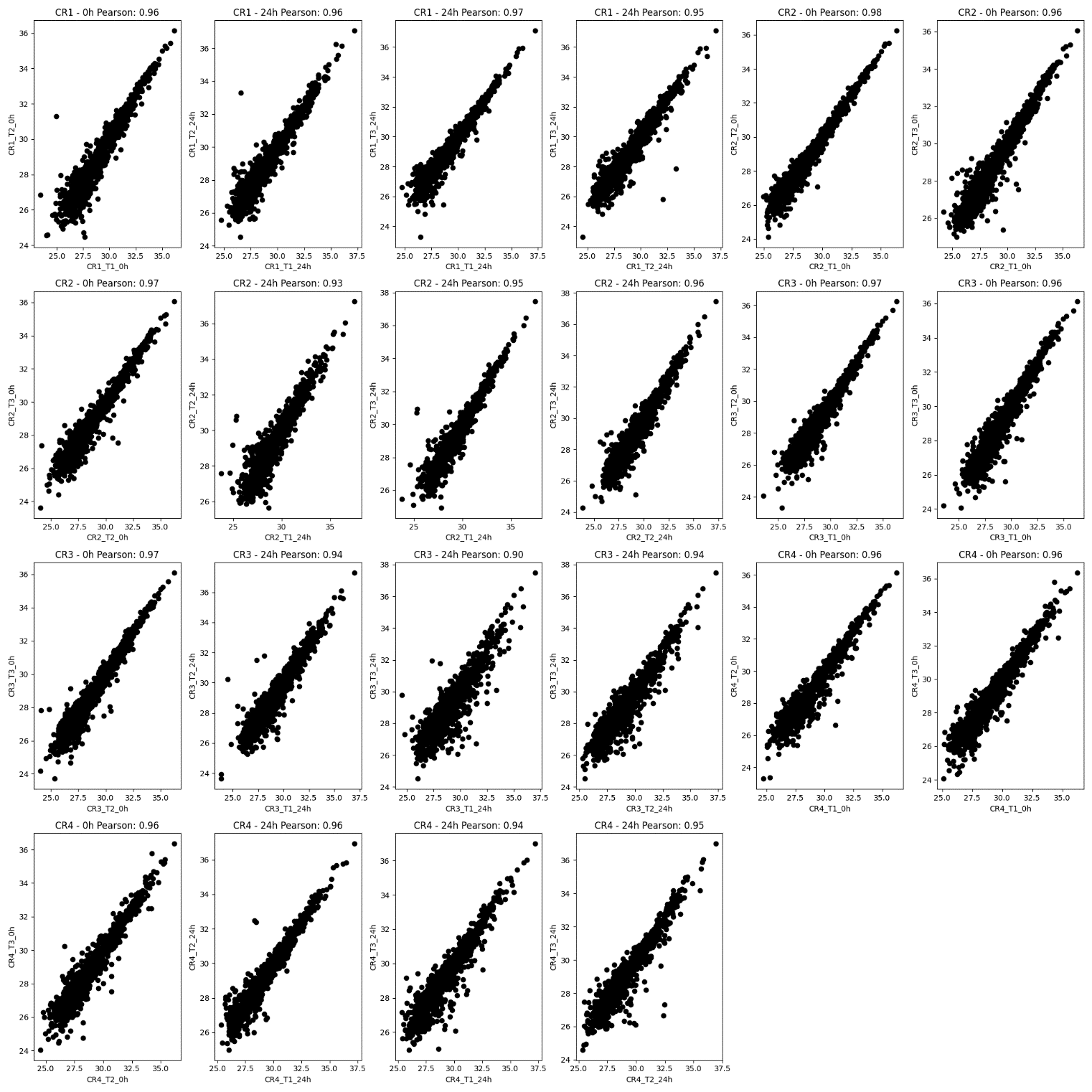


Figure S12. **Multi scatter plots for all technical replicates for the control and treatment samples in isolate 989a (AR, T-technical replicate).** The plots are generated by Python and Pearson correlation values are shown.

**References:**

Mojsoska, B., Zuckermann, R. N., & Jenssen, H. (2015). Structure-activity relationship study of novel peptoids that mimic the structure of antimicrobial peptides. *Antimicrobial Agents and Chemotherapy*, *59*(7), 4112–4120. https://doi.org/10.1128/AAC.00237-15
